# Editorial: Knowledge, attitudes and perceptions of healthcare professionals and health professions students towards vaccinations and non-pharmaceutical interventions

**DOI:** 10.3389/fmed.2024.1443129

**Published:** 2024-10-28

**Authors:** Marília Silva Paulo, Alberto Modenese

**Affiliations:** ^1^CHRC, NOVA Medical School, Faculdade de Ciências Médicas, NMS, Universidade Nova de Lisboa, Lisboa, Portugal; ^2^Department of Biomedical, Metabolic and Neural Sciences, University of Modena and Reggio Emilia, Modena, Italy

**Keywords:** non-pharmaceutical interventions, vaccines, vaccinations, infectious, risk perception, SARS-CoV-2, COVID-19, prevention

The COVID-19 pandemic has brought to the fore the importance of rigorous prevention of infectious risk inside and outside of healthcare workplaces: this Research Topic aims to provide new and up-to-date perspectives on the prevention of this risk, with a particular focus on healthcare workers (HCW) and health professions students (HPS). Among the main preventive interventions, vaccination campaigns and non-pharmaceutical interventions (NPIs) are fundamental (1). HCW and HPS have a specific background on the topics of infectious risk prevention, even if possibly more focused on patients' protection, rather than on occupational risk prevention, which may be seen as a corollary, not deserving further specific attentions. Nevertheless, scientific literature shows that HCW and HPS might present cultural barriers when adhering to the necessary procedures to protect themselves from the occupational infectious risk. These issues were known also before the pandemic, e.g. when observing the quite low rates of HCW and HPS vaccinated against influenza virus, highlighted in many Countries (2).

The study of Beltrán et al., conducted in Colombia on 307 HCWs during the COVID-19 pandemic, highlights the importance of raising the awareness of HCWs regarding infectious risk prevention at the workplaces. Among the relevant points raised by the Authors' work, it's worth noticing that infections in HCWs occurred not only through patient care, but also through exposure to close contacts with colleagues. Furthermore, the study shows a relevant improvement in occupational infectious risk prevention, together with the evolving of the pandemic and the progresses made in understanding and providing the most effective protections to the workers, including vaccinations. The Authors reported a decreasing trend of the rates of occupational infections (i.e. defined as “close contact at work”) for HCWs. At baseline, 5.5% of the HCWs attributed this route of exposure to the causality of the infection, while at the first follow-up after 45 days only the 4.6% of the participants indicated work exposure as the main cause, and after 60 days none attributed the new infectious cases to close contacts at work. These results represent an important indication in terms of effectiveness of occupational preventive interventions in reducing infectious rates. Data like those obtained from the study of Beltrán et al. are useful for education initiatives and occupational safety and health (OSH) trainings of HCW and HPS. In fact, these data provide practical demonstrations of occupational infectious risk reduction after the adoption of fundamental protective measures, for example, workplace sanitization, rigorous PPE use and vaccination.

Effective education and training of workers and students on these topics is extremely important: during the pandemic, various issues concerning inadequate habits and believes of HCW and HPS toward vaccinations and infectious risk prevention emerged. These issues were somewhat amplified by the so-called “infodemic” (3), spreading not only fundamental scientific knowledge, but sometimes also various non-scientifically based information, e.g., on vaccines' safety and on SARS-CoV-2 contagion characteristics. These latter aspects of the infodemic might have fueled the poor attitudes toward the application of recommended measures for the prevention of infectious risk and the resistances in promptly accepting COVID-19 vaccinations showed by a minority of the HCW and HPS. Well-designed OSH trainings need to provide them with the necessary cultural background, allowing a correct interpretation of the results of scientific research, to gain an evidence-based approach on occupational risks' prevention. Following such education programs, HCW and HPS would be able to acquire the instruments for reading and understanding the scientific data, including different effectiveness and risk–benefit ratios of the available preventive interventions. An additional way to have HCW and HPS more familiar with the principles of medical science and evidence-based prevention is to promote their direct involvement and participation in research activities. Alshamrani et al. show that radiology practitioners and interns in Saudi Arabia have a great interest in research, nevertheless only half of them actually have the time to participate in scientific studies and publications. This is a well-known and common problem for all the researchers involved in clinical activities: the results of Alshamrani et al. study are important, and can serve as a valuable basis for designing developmental programs aimed at overcoming research obstacles among HCW and HPS. Operators and students involved in research activities can achieve a better knowledge and a deeper understanding on how the results of scientific studies impact on the adoption of prevention practices at work.

Finally, as mentioned above, one of the lessons learned during the pandemic is that, in emergency situations, there can be an uncontrolled spread of news, many of them scientifically based, but possibly a part of them without the necessary evidence-based scientific basis. The general public may not have the scientific background allowing them to interpret whether an information is correct or not. During the pandemic we discovered that, unfortunately, even some HCW and HPS developed non-scientifically based interpretations of the ways to be followed for a proper infectious risk prevention. This is a serious problem, as HCW and HPS are responsible not only for protecting themselves, but their risky habits can pose additional hazards for colleagues and patients. Moreover, HCW are supposed to spread to the general public correct information on infectious risk prevention, directly resulting from the evidence-based process sustaining the publication of research data. The infodemic undermined this process in some cases, fortunately only in a minority of the research works published, but this was sufficient to determine a relevant issue: we will have to learn from this, and the acquisition of fundamental skills in interpreting research data and their sources will need to deserve a priority place in future education and OSH trainings of HCW and HPS. Actually, evidence-based prevention strategies to deal with the initial phases of SARS-CoV-2 epidemic were mainly applied in healthcare workplaces for the protection of HCW, HPS and their patients, but quickly the emergency acquired the dimensions of a pandemic and all the scientifically-based effective protections available needed to be rigorously applied also in the communities outside of the healthcare structures. A scientific based approach in evaluating the effectiveness of public health and social measures (PHSMs) applied to contain the spread of the SARS-CoV-2 virus among general public has been proposed within the systematic review of Paulo et al.: the results of the study show that the measures with highest evidence of positive impact are social distancing, hygiene measures, use of face masks and testing policies. Interestingly, based on a Delphi consensus approach, the study was able to give a higher or lower weight to each of these PHSM based on different time periods representing the evolution of the COVID-19 pandemic. The evidence found in this Systematic Review has significant implications, for both researchers and policymakers, that need to be considered in educational and preparedness programs and activities in the public health space. Proper information and specific training to make HCW and HPS, and therefore also the general public, able to understand when the adoption of protective measures could be considered effective are extremely important within such programs.

As a conclusion, the three articles published under the present Research Topic address different aspects of the infectious risk prevention at the healthcare workplaces, with repercussions also for the general population. Probably, one of the key messages that should be taken by the readers is that the scientific approach is fundamental not only for therapeutical interventions, but also for preventive interventions. The scientific evidence and research activities building the basis for the recommendations on infectious risk prevention need to be clearly communicated and included in educational programs and OSH trainings for all HCW and HPS, minimizing the risk of miscommunications: only the promotion of this approach can ensure for healthcare operators a correct adherence to preventive interventions and to vaccinations campaigns, and a proper adoption of the recommended protections.
